# Diabetic Cardiomyopathy: Impact of Biological Sex on Disease Development and Molecular Signatures

**DOI:** 10.3389/fphys.2018.00453

**Published:** 2018-05-03

**Authors:** Ryan Toedebusch, Anthony Belenchia, Lakshmi Pulakat

**Affiliations:** ^1^Cardiovascular Medicine Division, Department of Medicine, University of Missouri, Columbia, MO, United States; ^2^Dalton Cardiovascular Research Center, University of Missouri, Columbia, MO, United States; ^3^Department of Nutrition and Exercise Physiology, University of Missouri, Columbia, MO, United States

**Keywords:** cardiac hypertrophy, fibrosis, cardiovascular disease, diabetes, microRNA, estrogen, sex hormones

## Abstract

Diabetic cardiomyopathy refers to a unique set of heart-specific pathological variables induced by hyperglycemia and insulin resistance. Given that cardiovascular disease (CVD) is the leading cause of death in the world, and type 2 diabetes incidence continues to rise, understanding the complex interplay between these two morbidities and developing novel therapeutic strategies is vital. Two hallmark characteristics specific to diabetic cardiomyopathy are diastolic dysfunction and cardiac structural mal-adaptations, arising from cardiac cellular responses to the complex toxicity induced by hyperglycemia with or without hyperinsulinemia. While type 2 diabetes is more prevalent in men compared to women, cardiovascular risk is higher in diabetic women than in diabetic men, suggesting that diabetic women take a steeper path to cardiomyopathy and heart failure. Accumulating evidence from randomized clinical trials indicate that although pre-menopausal women have lower risk of CVDs, compared to age-matched men, this advantage is lost in diabetic pre-menopausal women, which suggests estrogen availability does not protect from increased cardiovascular risk. Notably, few human studies have assessed molecular and cellular mechanisms regarding similarities and differences in the progression of diabetic cardiomyopathy in men versus women. Additionally, most pre-clinical rodent studies fail to include female animals, leaving a void in available data to truly understand the impact of biological sex differences in diabetes-induced dysfunction of cardiovascular cells. Elegant reviews in the past have discussed in detail the roles of estrogen-mediated signaling in cardiovascular protection, sex differences associated with telomerase activity in the heart, and cardiac responses to exercise. In this review, we focus on the emerging cellular and molecular markers that define sex differences in diabetic cardiomyopathy based on the recent clinical and pre-clinical evidence. We also discuss miR-208a, MED13, and AT2R, which may provide new therapeutic targets with hopes to develop novel treatment paradigms to treat diabetic cardiomyopathy uniquely between men and women.

## Introduction

Cardiovascular diseases (CVDs) are the number one killer of both men and women worldwide (World Health Organization [WHO], 2017). Type 2 diabetes mellitus (T2DM) is an independent risk factor for CVD (Lotufo et al., 2001; Abdul-Ghani et al., 2017; Archundia Herrera et al., 2017; Davis et al., 2017). Diabetic patients have a two- to fourfold increased risk for CVD development, and undoubtedly, the increased prevalence of T2DM has led to more documented cases of cardiovascular complications (Kannel et al., 1974; Kannel and McGee, 1979; Martin-Timon et al., 2014; Bertoluci and Rocha, 2017). Indeed, while T2DM contributes to overt CVD, it has been documented to be responsible for a unique set of cardiac abnormalities (Miki et al., 2013; Kain and Halade, 2017; Varma et al., 2017), referred to as diabetic cardiomyopathy. Several of the cellular mechanisms documented to underlie the development of contractile dysfunction in diabetic cardiomyopathy include: impaired excitation-contraction coupling, inefficient energy production, reduced coronary flow reserve, and fibrotic remodeling (Miki et al., 2013). Not surprising, many of these mechanisms are also observed in hypertrophic and dilated cardiomyopathies, independent of diabetes, promoting the equivocation and questioning a universal clinical definition for diabetic cardiomyopathy. In further support of the ambiguity, a review by Holscher et al. (2016) reported that “instead of being a cardiomyopathy in the classical sense though, diabetic cardiomyopathy represents a combination of molecular myocardial abnormalities that predispose for the development of myocardial dysfunction.” Additional questions regarding diabetic cardiomyopathy and its progression in both insulin-dependent and insulin-independent diabetic patients remain unanswered. Although a ubiquitous definition has yet been agreed upon, diabetic cardiomyopathy is generally accepted to refer to diabetes-associated changes in the structure and function of the myocardium, independent of other peripheral disease, in otherwise healthy diabetic patients (Dandamudi et al., 2014).

The first documented associations between diabetes mellitus and any cardiovascular complications appeared in the first half of the 20th century (Liebow and Hellerstein, 1949). However, the first reports of what we know today as “diabetic cardiomyopathy” did not appear until the early 1970s when it was documented that diabetic patients with congestive heart failure, who had no evidence of coronary atherosclerosis, had abnormal fibrosis patterns within the myocardium (Rubler et al., 1972). Shortly after, the Framingham Study provided epidemiological data supporting the notion that a specific cardiomyopathy does exist in diabetics, and that cardiovascular mortality was threefold higher in participants with diabetes (Kannel et al., 1974). In the ∼40 years since these seminal studies appeared, increased incidence of T2DM, CVD, and the complex interactions between them have underscored the need for a better understanding of the pathology and possible treatments. Beyond epidemiological and associative data describing diabetic cardiomyopathy, the basic science and general understanding of its progression, incidence, and possible treatment(s) remain in their infancy. Nevertheless, mounting evidence suggests that the incidence of diabetic cardiomyopathy is sex and age dependent (Kautzky-Willer et al., 2016).

The Framingham Heart Study first revealed that diabetic women have a 5.1-fold increase in heart failure, while diabetic men only have a 2.4-fold increase compared to non-diabetic women and men, respectively (Kannel et al., 1974). Since that time, several studies provided similar evidence that diabetic women have a greater relative risk of CVD compared to diabetic men (Heyden et al., 1980; Barrett-Connor et al., 1991; Simons et al., 1996; Folsom et al., 1997; Natarajan et al., 2003). Laverty et al. (2017) assessed gender differences in hospital admissions for major cardiovascular events in diabetics throughout England over 10 years between 2004 and 2014. They confirmed that diabetic women, compared to men, had increased hospital admission rates for acute myocardial incident (AMI), percutaneous coronary intervention (PCI), and coronary artery bypass grafting (CABG). Specifically, compared to non-diabetic women, diabetic women had a 4.3-fold increase in AMI, 4.4-fold increase in PCI, and 6.2-fold increase in CABG admissions, supporting the notion that diabetic females have an elevated incidence of CVD risk factors, compared to diabetic men. Further, pre-menopausal women usually present with CVD almost a decade later than males; however, this protection is not seen in pre-menopausal women diagnosed with T2DM (Norhammar and Schenck-Gustafsson, 2013).

Considering unique sex-based differences in physiology and pathophysiology, it is worth noting some of the most apparent sex-specific factors that underlie our understanding of the above-mentioned observations. First, it has been documented that females have altered systemic glucose regulation, compared to males (Basu et al., 2006; Flanagan et al., 2006). For example, in one clinical study, it was shown that systemic insulin was higher after dextrose infusion in females, suggesting lower insulin-sensitivity at baseline in females compared to males (Flanagan et al., 2006). Second, pre-menopausal women demonstrate different fat distribution compared to men. In contrast to the central (visceral) adiposity observed in males, females tend to have peripheral fat distribution, contributing to higher insulin sensitivity at greater levels of body mass (Wannamethee et al., 2012). Due to this, women typically have increased metabolic disturbances (body mass index and insulin resistance) before overt T2DM diagnosis and therefore incur higher CVD risk (Logue et al., 2011; Gomez-Marcos et al., 2015). Third, and possibly the most important, yet commonly misunderstood, is the role that estrogen-related signaling within the female myocardium plays in disease progression and/or protection. Finally, a strong correlation is reported between the leukocyte telomere length (LTL) shortening and increase in metabolic syndrome components in females (Cheng et al., 2017). LTL shortening and low telomerase activity are shown to be associated with CVD, coronary artery disease, diabetes mellitus, cardiomyopathy, and all-cause mortality (Bar et al., 2014; Yeh and Wang, 2016; Sawhney et al., 2018). It is noteworthy that while telomerase activity in cardiomyocytes decreases in men with aging, it increases in women (Leri et al., 2000; Kajstura et al., 2010). However, how T2DM modulates this effect in the heart tissues of women versus men is currently unclear.

## Diabetic Cardiomyopathy and an Unresolved Definition

Scientists and clinicians alike face challenges when studying the pathophysiology of diabetic cardiomyopathy. Diabetic cardiomyopathy commonly presents with one or more comorbidities known to exacerbate heart failure. Functionally, diabetic cardiomyopath*y* is characterized by diastolic dysfunction, defined as a defect in left ventricular relaxation leading to increased pressures and a subsequent impaired filling during diastole (Lorenzo-Almoros et al., 2017). In both type 1 (T1DM) and T2DM, diastolic dysfunction is largely considered a hallmark of diabetic cardiomyopathy, although some reports suggest that upon adjustment for comorbidities, diastolic dysfunction is not statistically significant (Wachter et al., 2007; Fontes-Carvalho et al., 2015). Stahrenberg et al. (2010) demonstrated that along the continuum of diabetic patients, higher HbA_1c_ levels are associated with the severity of diastolic dysfunction, as measured by E/E′, a non-invasive estimate of left atrial filling pressure that independently predicts primary cardiac events.

**FIGURE 1:**
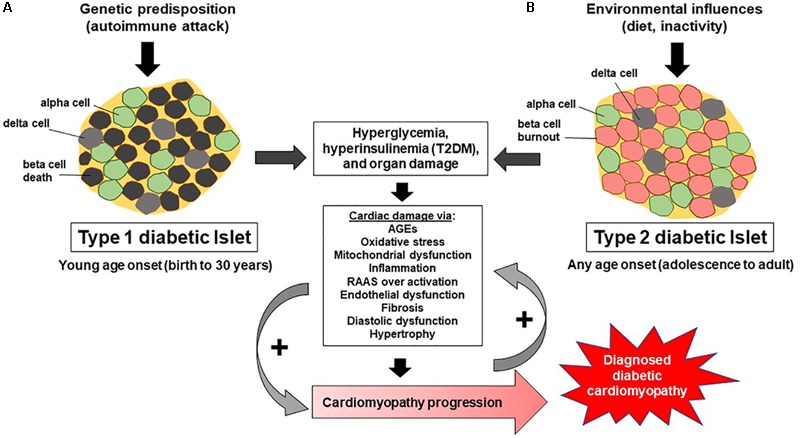
Progression of T1DM and T2DM and development of diabetic cardiomyopathy. **(A)** Illustrates how an immune mediated response in T1DM destroys beta cells within pancreatic islets, leading to hyperglycemia, resulting in cardiac damage which promotes the development of diabetic cardiomyopathy. **(B)** Shows how environmental influences (diet, inactivity) lead to insulin resistance, hyperinsulinemia, beta cell burnout, and subsequent dysregulation of glucose, leading to hyperglycemia, cardiac damage, and ultimately diabetic cardiomyopathy. Noteworthy is the arrow indicating the progression of diabetic cardiomyopathy and that it occurs and progresses uniquely in certain populations of individuals, namely male and females.

In diabetes, diastolic function as assessed by E/A ratio and hemodynamics has been shown to prematurely deteriorate compared to healthy controls. The E/A ratio represents the ratio of the E wave (peak blood flow velocity in early diastole) to the A wave (peak blood flow velocity in late diastole) caused by atrial contraction. The filling pattern, where there is a reduction in the E/A ratio along with prolongation of the deceleration time of E, indicates impaired relaxation. E/A values measured in young (20–32 years of age) T1DM males match that of healthy men at 50 years of age, suggesting that T1DM negatively effects diastolic function (Berkova et al., 2003). Two more recent reports (Jensen et al., 2014; Suran et al., 2016) using echocardiography evaluation of T1DM patients without known CVD, reported the presence of diastolic dysfunction. Indeed, one study assessed adolescent T1DM patients (mean duration of disease = 6 years) and found, both at rest and during exercise, these patients had reduced diastolic function (Gusso et al., 2012), evidenced by end diastolic volume (Holscher et al., 2016). In contrast, various other reports suggest that in long-term T1DM patients, evidence for diastolic dysfunction is lacking (Zarich et al., 1988; Romanens et al., 1999), indicating that T1DM’s ability to cause diastolic dysfunction may be a factor of duration of disease, age of onset, management, and/or environment. The deleterious effects of diabetes on myocardial parameters are not synonymous between patients with T1DM versus T2DM, adding to the vagueness of diabetic cardiomyopathy. For example, T1DM is mostly associated with hyperglycemia, oxidative stress, and resultant myocardial fibrosis and average patient population with T1DM is younger than that with T2DM (**Figure 1**). In contrast, T2DM is linked to hyperinsulinemia, insulin resistance, obesity, and cardiomyocyte hypertrophy (Lorenzo-Almoros et al., 2017).

**FIGURE 2:**
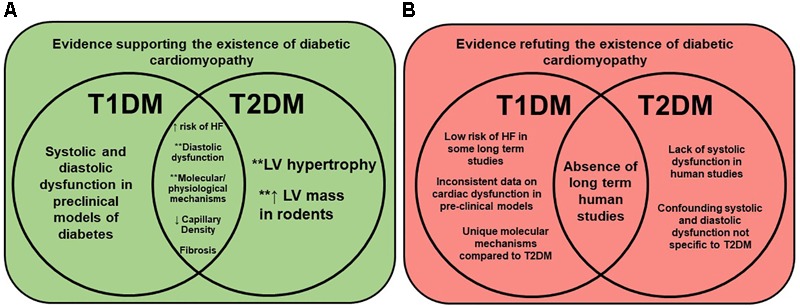
Evidence for and against the existence of diabetic cardiomyopathy. Venn diagrams show the current evidence for (Panel **A**, green) and against (Panel **B**, red) the existence of diabetic cardiomyopathy, and how they relate to T1DM, T2DM, and/or both. Molecular/physiological mechanisms documented in rodent models include oxidative stress, glucotoxicity, lipotoxicity, metabolic inflexibility, cardiac fibrosis, and mitochondrial dysfunction (Boudina et al., 2005; Schilling and Mann, 2014; Holscher et al., 2016). Diastolic dysfunction implies heart failure with either preserved (HFpEF) or reduced (HFrEF) ejection fraction. HF = heart failure, T1DM = type 2 diabetes, T2DM = type 2 diabetes, and LV = left ventricle. ^∗∗^Indicates possible sex difference documented in literature.

A 2003 publication assessed the fact that T1DM patients may present with diabetic autonomic neuropathy (DAN), and hypothesized that this may account for diastolic and/or systolic dysfunction (Didangelos et al., 2003). Using radionuclide ventriculography, it was found that T1DM patients had reduced diastolic parameters, including atrial contribution to ventricular filling, peak filling rate, first third filling fraction, and time to peak filling, suggesting diastolic dysfunction (Didangelos et al., 2003). The lack of cardiac dysfunction in long-term T1DM patients may also be related to permanent treatment with exogenous insulin. Others have suggested that myocardial overload and increased peripheral resistance resultant from exogenous insulin may be responsible for the observed diastolic dysfunction, rather than being symptoms of diabetic cardiomyopathy (Holscher et al., 2016). In summary, numerous reports support the fact that diastolic dysfunction is the defining characteristic of diabetic cardiomyopathy in both T1DM and T2DM patients. However, until additional long-term studies are performed, debate will continue until a universal definition is settled upon. **Figure 2** summarizes the evidence for and against the (current) understanding and existence of diabetic cardiomyopathy.

Alternatively, when considering rodent models of T1DM, clinical data regarding diastolic dysfunction has been corroborated (Bugger and Abel, 2009). On a cellular and molecular level, rodent models of diabetic cardiomyopathy have helped develop our understanding of myocardial alterations. Due to their resistance to atherosclerosis, they provide a good model to study the effects of diabetes on the myocardium, independent of peripheral complications (Severson, 2004). Through the use of rodent models, mechanisms underlying diabetic cardiomyopathy, including cardiac fibrosis, mitochondrial dysfunction, oxidative stress, lipotoxicity, and metabolic inflexibility have been identified (Schilling and Mann, 2014).

Importantly, there are a few human studies that lend support to rodent findings, including mitochondrial dysfunction, fibrosis, oxidative stress, and metabolic inflexibility (Peterson et al., 2004; Anderson et al., 2009), suggesting that rodent models may be a sufficient means to study diabetic cardiomyopathy progression. For example, consistent with observations from diabetic rat models, intra-myocardial lipid accumulation is seen in the failing heart of diabetic patients. Moreover, intra-myocardial lipid accumulation in T2DM patients correlates with impaired cardiomyocyte contractility and their inability to adapt after myocardial infarction, a phenomenon seen in diabetic rats (Sharma et al., 2004; Borisov et al., 2008). It was also reported that in obese, insulin-resistant men, abnormal left ventricular energy metabolism is observed prior to structural and functional pathological remodeling in the heart (Perseghin et al., 2007).

Together, these data further the potential uniqueness of diabetic cardiomyopathy in different models and buttress the existence of inherent limitations when comparing between clinical (human) and pre-clinical (rodent) experimental data. One feature that has long been known and is under no debate is that cardiomyocytes experience a loss of metabolic flexibility in the face of chronically high glucose in animal models of diabetes (Chatham and Forder, 1993; Stanley et al., 1997). The consequence of this on the progression of diabetic cardiomyopathy is important, yet our understanding of how this contributes to fibrotic remodeling and diastolic dysfunction remains to be fully understood. In this context, Galgani et al. (2008) reported that metabolic inflexibility to glucose in humans with T2DM is mostly related to defective glucose transport. One theme common to rodent and human studies is the disparity for the inclusion of female subjects (Miller and Best, 2011; Mozaffarian et al., 2015), resulting in a lack of data specific to sex differences and treatment options specific to females. The gender-specific differences in diabetic cardiomyopathy and its progression, at the level of the myocardium remain largely unknown. A purpose of this review is to highlight the known data on the human and animal literature interrogating molecular mechanisms and sex differences contributing to diabetic cardiomyopathy.

## Contribution of Sex Hormones in Diabetic Cardiomyopathy

Testosterone is the primary male sex hormone responsible for maturation of sex organs, sperm production, and secondary sexual characteristics in males (Kloner et al., 2016). It is well established that testosterone levels peak during the third decade of life (Gray et al., 1991), and then decline at a rate of 1–2% annually thereafter (Feldman et al., 2002). Epidemiological studies have shown that low levels of testosterone associate with increased risk of CVD (Rosano et al., 1999; Araujo et al., 2011; Corona et al., 2011; Ruige et al., 2011). Furthermore, one in three type-2 diabetic men are testosterone deficient, suggesting that low testosterone levels are associated with metabolic syndrome and T2DM (Spark, 2007). In a streptozotocin-induced model of T1DM using Sprague–Dawley rats, treatment with testosterone increased cardiac angiogenesis, highlighting the cardioprotective role of testosterone (Chodari et al., 2016). Given the associations between testosterone, aging, CVD, and metabolic diseases such as T2DM, further explorations into the possible role that testosterone levels play in the progression of diabetic cardiomyopathy are warranted. There is a paucity of data describing the specific effects of low testosterone levels on the development/progression of diabetic cardiomyopathy in males, or the underlying molecular and cellular mechanisms.

Estrogen, along with progesterone, are two female sexual-reproductive hormones that are precisely regulated throughout the life course. Estrogens (estrone, estriol, and the biologically active 17β-estradiol) are derived from cholesterol and produced via aromatization of androgens. Females, by virtue of higher estrogen levels, compared to males, have higher protection during pre-menopausal years on various organ systems, including cardiovascular and others (Karas et al., 2001; Mendelsohn, 2002). However, these protective effects are attenuated, and often times lost, when estrogen levels drop off significantly at the onset of menopause. In pre-menopausal women, serum estrogen concentrations range from 367 pmol per liter to 2200 pmol per liter. Upon menopause, these levels decrease significantly to between 18 and 74 pmol per liter, analogous to levels in similarly aged men (Yen and Jade, 1991). Free estrogen in the circulation binds to one of two receptors, estrogen receptor alpha (ER_α_) and estrogen receptor beta (ER_β_), both of which vary in expression depending on tissue type and age (Nilsson and Gustafsson, 2011; Vrtacnik et al., 2014).

In addition to binding to its own receptor and subsequent intracellular signaling, estrogen can affect various physiological functions, not limited to serum lipid concentrations, antioxidant systems, nitric oxide (NO) production, and coagulation pathways (Mendelsohn and Karas, 1999). Estrogen has rapid effects (non-genomic), such as increased NO production and vasodilation, as well as long-term effects (genomic), including decreased atherosclerosis, vascular injury, smooth muscle growth, and increased endothelial cell growth and smooth muscle differentiation (Mendelsohn and Karas, 1999; Wu et al., 2015). Endothelial and vascular smooth muscle cells both express estrogen receptors. Endothelial cells are the first line of defense against any foreign substance that remains in the blood stream and may contribute to vascular disease (Karas et al., 1994; Venkov et al., 1996; Gargett et al., 2002). *In vitro*, estrogen is capable of increasing NO release from endothelial cells rapidly, prior to any changes in gene expression (Caulin-Glaser et al., 1997; Lantin-Hermoso et al., 1997). Released NO signals to the vascular smooth muscle cells (VSMCs) to relax, contributing to vasodilation and inhibiting platelet activation (Benjamin et al., 1991; Sandoo et al., 2010). Endothelial nitric oxide synthase (eNOS) and neuronal nitric oxide synthase (nNOS), responsible for synthesizing NO, have also been shown to be regulated by endogenous estrogen levels. This suggests that the primary mediators of vascular tone, NOS and NO, are involved in the dysregulation and/or increased protective effects in both males and females, during the development of diabetic cardiomyopathy (Weiner et al., 1994), but are likely to play a more significant role in females. A New England Journal of Medicine article concludes “Direct myocardial effects of estrogen on cardiac structure and function are likely to be important as well and deserve greater attention” (Mendelsohn and Karas, 1999). In female rats, the absence of estrogen is associated with left ventricular hypertrophy, collagen deposition, and increased sensitivity to angiotensin II (Xu et al., 2003; Dean et al., 2005). Estrogen offers protection to cardiomyocytes via prevention of apoptosis and reduced infarct size (Olivetti et al., 1995; Patten et al., 2004; Kim et al., 2006).

Considering that one hallmark characteristic of diabetic cardiomyopathy is damage to the myocardium in the absence of atherosclerosis or peripheral artery disease, it is important to realize the effects of estrogen on the myocardium and cardiomyocytes. With the onset of menopause, and subsequent decrease in estrogen levels, many women begin hormone replacement therapy (HRT) in order to maintain higher physiological levels of estrogens. While this is not without side effects [see reviews for HRT negative consequences (Yang and Reckelhoff, 2011)], evidence from both prospective and retrospective observational studies show that HRT offers protection from CVD in previously healthy women by 35–50% (Grady et al., 1992; Mendelsohn and Karas, 1994; Grodstein et al., 1996, 1997; Barrett-Connor, 1997; Iorga et al., 2017). What is less clear are the many interactions between low levels of estrogen, CVD, and existing T2DM, and if HRT provides protection from diabetic cardiomyopathy in longstanding diabetics. Attempts to tease out the specific underlying mechanisms, whereby chronically high glucose damages the myocardium and the role estrogen may play in preventing this in females remain in their infancy and require further attention.

## Oxidative Stress and Diabetic Cardiomyopathy

Cardiac oxidative stress is thought to be one of the primary insults leading to subsequent fibrosis, apoptosis, cellular damage, and hypertrophy (Huynh et al., 2014). One study examined 12-week-old female Sprague–Dawley rats after induction of T1DM using Alloxan and reported that markers of oxidative stress were dysregulated in diabetic animals compared to age-matched control rats. The authors suggest that in their model, Alloxan-induced diabetes caused cardiomyopathic changes, and these changes are likely mediated by oxidative stress (Aksakal et al., 2011). Additional studies have documented the association between oxidative stress and T1DM-induced cardiac damage (Zhang et al., 2017). Similarly, pre-clinical models of T2DM have been used to assess the role of oxidative stress in the progression of diabetic cardiomyopathy, with several transgenic, and diet-induced models suggesting a strong connection between diabetic cardiomyopathy and oxidative stress (Zhang et al., 2010; Calligaris et al., 2013; Fuentes-Antras et al., 2015; Chong et al., 2017). Evidence suggests that oxidative stress exhibits sex differences. For example, superoxide dismutase activity was found to be higher in female rat heart compared to male rat heart indicating that oxidative stress is lower in female rat heart compared to male rat heart (Barp et al., 2002). In humans, *in vivo* biomarkers of oxidative stress are reported to be higher in young men than in women of the same age (Ide et al., 2002). Furthermore, reactive oxygen species (ROS) production in the vascular cells from males are higher than cells from females (Matarrese et al., 2011). Sex differences in oxidative stress are also seen in healthy term neonates and their mothers (Diaz-Castro et al., 2016). Female-specific reduced systemic oxidative stress may play a role in improved cardio-protection in healthy females compared to males.

Studies in db/db mice show that while both males and females exhibited age-associated increases in the left ventricular atherosclerosis biomarker, plasminogen activator inhibitor 1 (PAI-1), females had a marked increase as diabetes progressed, compared to males, indicating a possible role for PAI-1 in diabetic cardiomyopathy (Zhao R. et al., 2013; Bowden et al., 2015). Bowden et al. (2015) demonstrated that many of the deleterious diabetic complications were exacerbated in female db/db mice compared to their male counterparts, lending support to the hypothesis that diabetic women are more susceptible to diabetic cardiomyopathy (Peters et al., 2014). Surprisingly, LV fibrosis and SERCA2a expression were higher in diabetic mice, compared to controls, but did not show a sex-specific effect. However, in these same animals, oxidative stress was greater in db/db females compared to males (Bowden et al., 2015).

Cardiac fibrosis, which occurs as a by-product of oxidative stress, is a hallmark feature of CVD. In the diabetic heart, fibrosis has been documented to progress in the left ventricle as a result of increased expression and presence of collagens and other extracellular matrix proteins, which stiffens the ventricular walls (Wu et al., 2000; Asbun and Villarreal, 2006; Huynh et al., 2012). As a result, it is common for compensatory left ventricular hypertrophy to occur due to the heart attempting to compensate for increased stiffness. Clinical studies using cardiovascular magnetic resonance have shown significant sex differences in left ventricular remodeling and myocardial fibrosis among patients with heart disease and women show higher New York Heart Association (NYHA) class and greater left ventricular remodeling index (Chen et al., 2015; Li et al., 2017). The population of patients in this study included similar numbers of male and female patients with diabetes. Another study showed that women have higher levels of fibrosis than men among patients with long-standing persistent atrial fibrillation (Li et al., 2017).

## Human Data Supporting Sex Differences in Diabetic Cardiomyopathy

The rates of T2DM incidence are roughly 6.6 and 5.9% of the population for men and women, respectively (Centers for Disease Control and Prevention, 2017). Interestingly, diabetic cardiomyopathy incidence remains higher in diabetic females compared to diabetic males, and this is further complicated by the pre- and post-menopausal stages of women and the vital role that estrogen may be playing in disease protection and progression. A 2014 meta-analysis that analyzed 64 cohorts, including 858, 506 individuals, and 28,203 cardiac events, concluded that diabetic women have a 40% greater risk of incident coronary heart disease compared with diabetic men (Peters et al., 2014). Note that most of the studies to date focus on coronary heart disease rather than diabetic cardiomyopathy – implying that the participants were not free from peripheral disease, including hypertension, coronary artery disease and/or atherosclerosis. This is due to the absence of large cohort studies specifically looking at diabetic cardiomyopathy and sex differences. Additionally, virtually no large-scale human studies have inquired specifically into any mechanistic understanding of diabetic cardiomyopathy and the role that sex and sex hormones play.

A 2015 population-based survey examined 2,042 randomly selected residents in Minnesota, aged 45 years or older (Dandamudi et al., 2014). Participants underwent initial echocardiographic assessment of systolic and diastolic function and were followed for 3 years. It was reported that the incidence of diabetic cardiomyopathy in diabetic patients in this cohort was 16.9% and the prevalence of diastolic dysfunction was 54%. Indeed, the authors demonstrated that diabetic cardiomyopathy is associated with a high cumulative probability of the development of heart failure and death. The authors concluded that diabetic cardiomyopathy is relatively common in the studied community and that morbidity and mortality in patients with diabetic cardiomyopathy is high, reaching 31% in the studied population. Notably, this survey did not look at the differences between males and females, and more specifically, the role that sex hormones play in the incidence and progression of diabetic cardiomyopathy.

The Rancho Bernardo Study is a prospective population-based study of older adult inhabitants of Southern California. Subjects were recruited and monitored yearly with questionnaires and every 4 years by clinical evaluation. The study evaluated both male and female T2DM patients for 14 years and, among other outcome measures, concluded that “diabetes in women overrides their natural advantages and that is not entirely...mediated by many other conventional heart disease risk factors.” One aspect of this natural advantage that they discuss is the levels of high-density lipoprotein cholesterol (HDLC) being lower in diabetic women compared with both healthy women and diabetic men (Barrett-Connor et al., 1991). Furthermore, data suggests that HDLC at normal or elevated levels confers more protection in women than men (Crouse, 1989; Gordon et al., 1989).

## Rodent Data Supporting Sex Differences in Diabetic Cardiomyopathy

Our understanding of diabetic cardiomyopathy incidence in humans has largely been a result of the previously mentioned epidemiological and cross-sectional studies, and meta-analysis. However, the current understanding of diabetic cardiomyopathy progression and the mechanisms involved are largely a result of pre-clinical rodent studies. Several experimental rodent models have been developed to study various contributing factors to diabetic cardiomyopathy; including several genetically modified models; db/db mice, ob/ob mice, Otsuka Long-Evans Tokushima (OLETF) rats, CIRKO (cardiomyocyte deletion of insulin receptor) mice, cardiac lipotoxic mice [cardiomyocyte-specific long-chain acyl-CoA synthetase (ACS) and fatty acid transport protein (FATP1) overexpressing], Zucker diabetic fatty (ZDF), Zucker obese (ZO), lean (ZL) rats, and various other diet-induced obesity (DIO) strains. Monogenic, polygenic, and pharmacologically altered rodent models all provide insight into disease progression but are not without challenges when comparing back to human disease. Indeed, human diabetic cardiomyopathy is the result of a sequence of deleterious events that occur in succession, leading to eventual cardiac dysfunction in diabetic patients. The mechanisms elucidated from rodents and currently thought to be responsible for diabetic cardiomyopathy progression include: increased myocardial lipotoxicity, hypertrophy, decreased cardiac function, altered cytokine profile, increased oxidative stress, interstitial fibrosis, contractile and mitochondrial dysfunction, and altered myocardial metabolism.

The db/db and ob/ob genetic mouse models, both of which have altered leptin signaling and become obese, insulin-resistant and mild to severely hyperglycemic, provide a useful model to look at the damaging effects of diabetes on the myocardium. Noteworthy, few studies have included both males and females in an attempt to understand sex differences in these models. The db/db mice myocardium is characterized by increased oxidative stress, [(Boudina et al., 2007) male mice], cardiomyocyte apoptosis (Barouch et al., 2006), diastolic dysfunction [(Semeniuk et al., 2002) – male mice], and mitochondrial ROS [(Boudina et al., 2007) – male mice]. Isolated db/db hearts exhibit decreased left ventricular function compared to ob/ob hearts (Mazumder et al., 2004; Hafstad et al., 2006). Indeed, ob/ob mice have a reduction in ‘cardiac metabolic flexibility,’ meaning that in times of stress, i.e., hypoxia or ischemia, when a healthy heart would switch from fatty acids to glucose for fuel, obese ob/ob hearts are unable to switch substrate utilization (Boudina et al., 2005). Cardiac metabolism in db/db and ob/ob mouse models demonstrate decreased glucose oxidation and increased fatty acid oxidation. Indeed, this attenuation of metabolic flexibility increases the risk of ischemic damage and associates with insulin resistance and reductions in mitochondrial oxidative capacity.

The male ZDF rat has been extensively used as a rodent model of hyperglycemia and T2DM. Young ZDF-male rats exhibit severe hyperglycemia, diastolic dysfunction with preserved ejection fraction, reduced cardiac capillary density and cardiac structural damage caused by fibrosis and mitochondrial disorganization (Baynes and Murray, 2009; Pulakat et al., 2011). Diabetic cardiomyopathy in young hyperglycemic ZDF-female rats shares the features of reduced capillary density and mitochondrial damage with ZDF-male rats (Lum-Naihe et al., 2017). However, ZDF-female rats did not exhibit cardiac fibrosis, but demonstrated increase in heart weight and cardiomyocyte hypertrophy and higher levels of phosphorylation of cardiac mTOR (mechanistic target of rapamycin).

Myocardial contractility and structural parameters of the heart are different between male and female rats. Specifically, cardiomyocytes from male Wistar rats exhibit hypertrophy and undergo a greater degree of postnatal growth compared to female rats (Capasso et al., 1983). Similarly, 5-month-old ZL-male rats have larger hearts compared to ZL-female rats. Presence of T2DM is associated with increased risk of left ventricular hypertrophy, however, prolonged T2DM and severe hyperglycemia causes muscle loss. The 5-month-old ZDF-female rats that exhibited both hyperinsulinemia and hyperglycemia showed a greater degree of hypertrophy, both in terms of cardiomyocyte size and total heart size (adjusted to tibia length), compared to their lean counterparts, whereas age-matched ZDF male rats that had prolonged hyperglycemia did not show cardiac hypertrophy (Lum-Naihe et al., 2017). Blenck et al. (2016) have compared the sexual dimorphism in human and rodent cardiovascular physiology in a recent review.

**FIGURE 3:**
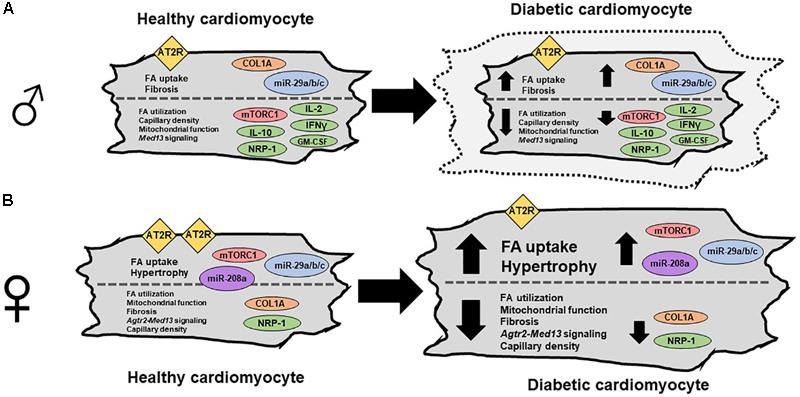
Sex differences in cardiomyocytes between healthy and diabetic cardiomyopathy. **(A)** Diagram of a male cardiomyocyte as it progresses from healthy to diabetic cardiomyopathy. In male, mTORC1 activation and hypertrophy are observed in some models but not others (indicated by dashed outer cardiomyocyte outline), while AT2R expression is not changed. While fatty acid (FA) uptake, fibrosis, collagen 1a, and miR-29a/b/c/ are increased with disease progression, the following are all reduced with disease progression; FA utilization, capillary density, mitochondrial function, and *Med13*-signaling, and several cytokines including IL-2, IL-10, IFN-γ, and GM-CSF. **(B)** Diagram of a female cardiomyocyte as it progresses from healthy to diabetic cardiomyopathy. In the female, mTORC1 activation and hypertrophy are observed. Additionally, miR-208a and miR-29a/b/c are increased, while FA utilization, mitochondrial function, fibrosis, *Agtr2-Med13* signaling, capillary density, collagen 1a, and NRP-1 are decreased (Widdop et al., 2003; Miki et al., 2013; Schilling and Mann, 2014; Lum-Naihe et al., 2017).

Previous studies have shown that there is a pan suppression of intracardiac cytokines in another T2DM rat model, the ZO-male rat (Luck et al., 2017). The ZO-male rat also has a mutation in the leptin receptor and is characterized by obesity, hyperinsulinemia, mild hyperglycemia, diastolic dysfunction with preserved ejection fraction, cardiac hypertrophy, and fibrosis (Gul et al., 2015; Luck et al., 2017). Compared to ZL-males, both ZO-male and ZDF-male rats exhibited a suppression of GM-CSF, IL-10, and IFN-γ (Luck et al., 2017; Lum-Naihe et al., 2017). GM-CSF, IL-10 (Wilson et al., 2007; Shi et al., 2014), and IFN-γ (Oldroyd et al., 1999; Emmez et al., 2008) are anti-fibrotic cytokines and their suppression could have contributed to cardiac fibrosis in these male T2DM models. Conversely, expression of these anti-fibrotic intracardiac cytokines was comparable between ZDF-female rats and ZL-female rats. This sex difference in the expression of anti-fibrotic intracardiac cytokines may be one of the reasons underlying the sex differences in diabetic cardiomyopathy (**Figure 3**). Interestingly, suppression of neuropilin-1, associated with increased cardiomyopathy (Wang et al., 2015), was observed in healthy females compared to male rats and also in diabetic rats of both sexes, compared to their healthy counterparts. This observation suggests that reduction in neuropilin-1 is an important contributor to diabetic cardiomyopathy.

Noteworthy is the sex difference in young ZDF-males and females in regards to DIO and T2DM (Mulder et al., 2010). ZDF-males develop hyperinsulinemia, hyperglycemia, and T2DM on standard chow. ZDF-females require a high fat diet with 48 kcal% Lard (Diet #12468 formulated by Edward A. Ulman, Ph.D. Research Diets, Inc.), in order to induce metabolic disease and T2DM. Thus, ZDF females need an additional insult, a high fat diet to induce T2DM. Importantly, this leads to the speculation that, in this model, females possess some level of “protection” and with it, an ability to delay hyperglycemia, hyperinsulinemia, and T2DM. Interestingly, the effect of DIO on the onset of T2DM differs also in mice and this effect depends on the age at which the high fat diet is started. In juvenile mice, DIO induces T2DM in males, but not in females (Salinero et al., 2018). However, if the DIO is initiated in adult mice, this sex difference in the induction of T2DM disappears.

Unlike the above rodent models that are characterized by systemic metabolic disruption, other models have been created that are cardiac specific and allow the deleterious effects of prolonged diabetes to be produced quickly in cardiac tissue. These models include CIRKO mice and cardiac lipotoxic mice (ACS and FATP1 overexpressing). The CIRKO mouse was first characterized in 2002 and provided a model that separated the intrinsic defects in cardiomyocytes from the potential confounding effects of altered systemic hyperglycemia (Belke et al., 2002). Similar to the CIRKO mouse, models of cardiac myocyte-specific liptoxicity provide an alternative lens to look at how fatty acid accumulation damages the heart muscle independent of peripheral and systemic disturbances. There have been two models developed that rely on excess fat entry into cardiomyocytes, mimicking severe diabetic cardiomyopathies. These models include cardiomyocyte-specific ACS and FATP1 overexpressing mice (Chiu et al., 2001, 2005). The resultant pathology exhibited large increases in fatty acids within the myocardium, and therefore, many of the deleterious effects on cardiac function are seen as in diabetic cardiomyopathy. These perturbations in lipid homeostasis within cardiomyocytes offers an experimental model to recapitulate the negative cellular effects commonly observed in clinical diabetic cardiomyopathy. However, these studies did not explore how biological sex differences modulate cardiac pathology of these models.

## Emerging Molecular Markers of Sex Differences in Diabetic Cardiomyopathy

Given the uniqueness of the molecular signature of diabetic cardiomyopathy, development and understanding of the targets and specific sex differences remains a vitally important area of inquiry. One of the molecules that regulates cardiac conduction and hypertrophy is the microRNA (miRNA) miR-208a (Callis et al., 2009). Interestingly, miR-208a exhibits sex differences, with expression being higher in the heart tissues of ZL-female rats compared to ZL-male rats (Lum-Naihe et al., 2017). T2DM increased miR-208a expression in both ZDF-male and ZDF-female hearts, resulting in ZDF-females having the highest expression. Thus miR-208a is a miRNA that links T2DM and cardiac dysfunction and hypertrophy. Notably, *ex vivo* studies on mouse aorta showed that miR-208a was suppressed by estrogen (Zhao J. et al., 2013). However, in healthy female rats with intact ovary, cardiac miR-208a expression was higher than that seen in males, indicating that estrogen-mediated regulation of miR-208a *in vivo* is different. A recent review by Florijn et al. (2017) discusses sex bias in miRNA expression and its correlation with the higher prevalence of heart failure with preserved ejection fraction (HFpEF) in women. HFpEF is a hall mark of diabetic cardiomyopathy. Pre-clinical studies in healthy mice and *in vitro* studies using estrogen have identified miRNAs either regulated by estrogen (miR-203, miR-126, miR-23a, miR-21, miR-24, miR-27a and b, and miR-106a and b) or encoded by X-chromosome (miR-98, miR-652, miR-221, miR-222, miR-223, miR-361, miR-421, miR-325, miR-188, miR-92a, miR-424, miR-503, miR-505). Authors suggest that estrogen-mediated regulation and/or link to X-chromosome play a role in sex bias of these miRNAs that contribute to CVD (Florijn et al., 2017).

Cardiac Mediator Complex 13 (MED13), encoded by the *Med13* gene, plays a critical role in systemic energy homeostasis and confers resistance to weight gain (Grueter et al., 2012). MED13 also improves systemic insulin sensitivity and glucose tolerance and is increased by treatment with mTOR complex 1 inhibitors rapamycin and nebivolol (Gul et al., 2015). Heart tissues of ZL-females exhibit higher levels of *Med13* mRNA compared to ZL-male rats, indicating that MED13 is a molecule that may provide increased cardioprotection in healthy female rat hearts. *Med13* mRNA is an established target of miR-208a (Grueter et al., 2012; Gul et al., 2015). T2DM suppressed cardiac *Med13* mRNA levels in both sexes, which is consistent with the T2DM-induced increase in miR-208a. Notably, both miR-208a and *Med13* expression levels were higher in ZL-female rats, indicating that in healthy female rats, other mechanisms contributed to increased expression of *Med13*; however, this advantage was lost in diabetic ZDF-female rats.

Angiotensin II type 2 receptor (AT2R), encoded by the X-linked *Agtr2* gene, has been documented to confer cardiovascular protective and reparative effects. Activating AT2R signaling by agonists and increasing *Agtr2* gene copy number by genetic manipulation in murine models improves cardiac repair and enhances cardiac function (Altarche-Xifro et al., 2009; Qi et al., 2012; Skorska et al., 2015). AT2R exhibits sex differences in cardiovascular expression (Okumura et al., 2005; Sampson et al., 2008; Hilliard et al., 2012; Lum-Naihe et al., 2017). AT2R expression is higher in the vasculature of healthy murine female models. This female-specific increased AT2R expression contributes to their increased resistance to hypertension and protection from cardiovascular and renal injury compared to males.

## Conclusion and Future Directions

The NIH (Clayton and Collins, 2014) has long recognized sex disparities in human clinical trials as a problem, and in 1993, passed the NIH Revitalization Act. In the 2 decades since, over half of NIH-funded clinical research participants are now women. The area that is still experiencing sex bias are pre-clinical studies, including *in vitro* and *in vivo* studies. In fact, a 2010 Nature opinion article shows that males still dominate animal studies (Zucker and Beery, 2010). Based on current trends, diabetes incidence will continue to increase and with it the co-morbidities, such as obesity, hypertension, and diabetic cardiomyopathy. Given the unique sex differences observed between males and females, future work needs to be aimed at understanding the role of sex-specificity on the progression of diabetic cardiomyopathy. There is desperately the need for a push for possible unique therapeutic treatment options that will address sex differences. Pre-clinical models can continue to help identify, test, and optimize possible therapies, with the hopes to make the leap into human trials. Paramount to this is the need for a continued scientific and medical discussion and elucidation of an agreed upon clinical definition of diabetic cardiomyopathy. Although the role of estrogen-mediated signaling in better cardiovascular protection of healthy females compared to males is well established, this female advantage is lost in T2DM and diabetic cardiomyopathy. Therefore, identifying additional therapeutic targets to improve cardiovascular outcomes of diabetic females is a critical need. AT2R and MED13 are cardioprotective molecules that exhibit female-specific increased expression in rodent studies. Moreover, currently there are no AT2R agonists in clinic to treat heart disease. Developing drugs that can increase expression of MED13 and AT2R in diabetic heart can lead to better treatment paradigms for diabetic cardiomyopathy and to mitigate sex differences in this pathology.

## Author Contributions

RT wrote the manuscript. RT, AB, and LP discussed the literature and figures, contributed to the intellectual input, and edited the manuscript. RT, AB, and LP approved the final version.

## Conflict of Interest Statement

The authors declare that the research was conducted in the absence of any commercial or financial relationships that could be construed as a potential conflict of interest.

## References

[B1] Abdul-Ghani M. A. Jayyousi A. DeFronzo R. A. Asaad N. Al-Suwaidi J. (2017). Insulin resistance the link between T2DM and CVD: basic mechanisms and clinical implications. *Curr. Vasc. Pharmacol.* 10.2174/1570161115666171010115119 [Epub ahead of print]. 29032755

[B2] Aksakal E. Akaras N. Kurt M. Tanboga I. H. Halici Z. Odabasoglu F. (2011). The role of oxidative stress in diabetic cardiomyopathy: an experimental study. *Eur. Rev. Med. Pharmacol. Sci.* 15 1241–1246. 22195355

[B3] Altarche-Xifro W. Curato C. Kaschina E. Grzesiak A. Slavic S. Dong J. (2009). Cardiac c-kit+AT2+ cell population is increased in response to ischemic injury and supports cardiomyocyte performance. *Stem Cells* 27 2488–2497. 10.1002/stem.171 19591228

[B4] Anderson E. J. Kypson A. P. Rodriguez E. Anderson C. A. Lehr E. J. Neufer P. D. (2009). Substrate-specific derangements in mitochondrial metabolism and redox balance in the atrium of the type 2 diabetic human heart. *J. Am. Coll. Cardiol.* 54 1891–1898. 10.1016/j.jacc.2009.07.031 19892241 PMC2800130

[B5] Araujo A. B. Dixon J. M. Suarez E. A. Murad M. H. Guey L. T. Wittert G. A. (2011). Clinical review: endogenous testosterone and mortality in men: a systematic review and meta-analysis. *J. Clin. Endocrinol. Metab.* 96 3007–3019. 10.1210/jc.2011-1137 21816776 PMC3200249

[B6] Archundia Herrera M. C. Subhan F. B. Chan C. B. (2017). Dietary patterns and cardiovascular disease risk in people with type 2 diabetes. *Curr. Obes. Rep.* 6 405–413. 10.1007/s13679-017-0284-5 29063379

[B7] Asbun J. Villarreal F. J. (2006). The pathogenesis of myocardial fibrosis in the setting of diabetic cardiomyopathy. *J. Am. Coll. Cardiol.* 47 693–700. 10.1016/j.jacc.2005.09.050 16487830

[B8] Bar C. Bernardes de Jesus B. Serrano R. Tejera A. Ayuso E. Jimenez V. (2014). Telomerase expression confers cardioprotection in the adult mouse heart after acute myocardial infarction. *Nat. Commun.* 5:5863. 10.1038/ncomms6863 25519492 PMC4871230

[B9] Barouch L. A. Gao D. Chen L. Miller K. L. Xu W. Phan A. C. (2006). Cardiac myocyte apoptosis is associated with increased DNA damage and decreased survival in murine models of obesity. *Circ. Res.* 98 119–124. 10.1161/01.RES.0000199348.10580.1d 16339484

[B10] Barp J. Araujo A. S. Fernandes T. R. Rigatto K. V. Llesuy S. Bello-Klein A. (2002). Myocardial antioxidant and oxidative stress changes due to sex hormones. *Braz. J. Med. Biol. Res.* 35 1075–1081. 10.1590/S0100-879X2002000900008 12219179

[B11] Barrett-Connor E. (1997). Sex differences in coronary heart disease. Why are women so superior? The 1995 Ancel Keys Lecture. *Circulation* 95 252–264. 10.1161/01.CIR.95.1.252 8994444

[B12] Barrett-Connor E. L. Cohn B. A. Wingard D. L. Edelstein S. L. (1991). Why is diabetes mellitus a stronger risk factor for fatal ischemic heart disease in women than in men? The Rancho Bernardo Study. *JAMA* 265 627–631. 10.1001/jama.1991.03460050081025 1987413

[B13] Basu R. Dalla Man C. Campioni M. Basu A. Klee G. Toffolo G. (2006). Effects of age and sex on postprandial glucose metabolism: differences in glucose turnover, insulin secretion, insulin action, and hepatic insulin extraction. *Diabetes Metab. Res. Rev.* 55 2001–2014. 10.2337/db05-1692 16804069

[B14] Baynes J. Murray D. B. (2009). Cardiac and renal function are progressively impaired with aging in Zucker diabetic fatty type II diabetic rats. *Oxid. Med. Cell. Longev.* 2 328–334. 10.4161/oxim.2.5.9831 20716921 PMC2835922

[B15] Belke D. D. Betuing S. Tuttle M. J. Graveleau C. Young M. E. Pham M. (2002). Insulin signaling coordinately regulates cardiac size, metabolism, and contractile protein isoform expression. *J. Clin. Invest.* 109 629–639. 10.1172/JCI13946 11877471 PMC150890

[B16] Benjamin N. Dutton J. A. Ritter J. M. (1991). Human vascular smooth muscle cells inhibit platelet aggregation when incubated with glyceryl trinitrate: evidence for generation of nitric oxide. *Br. J. Pharmacol.* 102 847–850. 10.1111/j.1476-5381.1991.tb12264.x 1906768 PMC1917966

[B17] Berkova M. Opavsky J. Berka Z. Skranka V. Salinger J. (2003). Left ventricular diastolic filling in young persons with type 1 diabetes mellitus. *Biomed. Pap. Med. Fac. Univ. Palacky.Olomouc Czech. Repub.* 147 57–61. 10.5507/bp.2003.008 15034606

[B18] Bertoluci M. C. Rocha V. Z. (2017). Cardiovascular risk assessment in patients with diabetes. *Diabetol. Metab. Syndr.* 9:25. 10.1186/s13098-017-0225-1 28435446 PMC5397821

[B19] Blenck C. L. Harvey P. A. Reckelhoff J. F. Leinwand L. A. (2016). The importance of biological sex and estrogen in rodent models of cardiovascular health and disease. *Circ. Res.* 118 1294–1312. 10.1161/CIRCRESAHA.116.307509 27081111 PMC4834858

[B20] Borisov A. B. Ushakov A. V. Zagorulko A. K. Novikov N. Y. Selivanova K. F. Edwards C. A. (2008). Intracardiac lipid accumulation, lipoatrophy of muscle cells and expansion of myocardial infarction in type 2 diabetic patients. *Micron* 39 944–951. 10.1016/j.micron.2007.11.002 18093836

[B21] Boudina S. Sena S. O’Neill B. T. Tathireddy P. Young M. E. Abel E. D. (2005). Reduced mitochondrial oxidative capacity and increased mitochondrial uncoupling impair myocardial energetics in obesity. *Circulation* 112 2686–2695. 10.1161/CIRCULATIONAHA.105.554360 16246967

[B22] Boudina S. Sena S. Theobald H. Sheng X. Wright J. J. Hu X. X. (2007). Mitochondrial energetics in the heart in obesity-related diabetes: direct evidence for increased uncoupled respiration and activation of uncoupling proteins. *Diabetes Metab. Res. Rev.* 56 2457–2466. 10.2337/db07-0481 17623815

[B23] Bowden M. A. Tesch G. H. Julius T. L. Rosli S. Love J. E. Ritchie R. H. (2015). Earlier onset of diabesity-Induced adverse cardiac remodeling in female compared to male mice. *Obesity* 23 1166–1177. 10.1002/oby.21072 25959739

[B24] Bugger H. Abel E. D. (2009). Rodent models of diabetic cardiomyopathy. *Dis. Model Mech.* 2 454–466. 10.1242/dmm.001941 19726805

[B25] Calligaris S. D. Lecanda M. Solis F. Ezquer M. Gutierrez J. Brandan E. (2013). Mice long-term high-fat diet feeding recapitulates human cardiovascular alterations: an animal model to study the early phases of diabetic cardiomyopathy. *PLoS One* 8:e60931. 10.1371/journal.pone.0060931 23593350 PMC3623942

[B26] Callis T. E. Pandya K. Seok H. Y. Tang R. H. Tatsuguchi M. Huang Z. P. (2009). MicroRNA-208a is a regulator of cardiac hypertrophy and conduction in mice. *J. Clin. Invest.* 119 2772–2786. 10.1172/JCI36154 19726871 PMC2735902

[B27] Capasso J. M. Remily R. M. Smith R. H. Sonnenblick E. H. (1983). Sex differences in myocardial contractility in the rat. *Basic Res. Cardiol.* 78 156–171. 10.1007/BF01906669 6870744

[B28] Caulin-Glaser T. Garcia-Cardena G. Sarrel P. Sessa W. C. Bender J. R. (1997). 17 beta-estradiol regulation of human endothelial cell basal nitric oxide release, independent of cytosolic Ca2+ mobilization. *Circ. Res.* 81 885–892. 10.1161/01.RES.81.5.885 9351464

[B29] Centers for Disease Control and Prevention (2017). *National Diabetes Statistics Report 2017.* Atlanta, GA: Centers for Disease Control and Prevention.

[B30] Chatham J. C. Forder J. R. (1993). A 13C-NMR study of glucose oxidation in the intact functioning rat heart following diabetes-induced cardiomyopathy. *J. Mol. Cell Cardiol.* 25 1203–1213. 10.1006/jmcc.1993.1133 8263954

[B31] Chen Y. Z. Qiao S. B. Hu F. H. Yuan J. S. Yang W. X. Cui J. G. (2015). Left ventricular remodeling and fibrosis: sex differences and relationship with diastolic function in hypertrophic cardiomyopathy. *Eur. J. Radiol.* 84 1487–1492. 10.1016/j.ejrad.2015.04.026 26001434

[B32] Cheng Y. Y. Kao T. W. Chang Y. W. Wu C. J. Peng T. C. Wu L. W. (2017). Examining the gender difference in the association between metabolic syndrome and the mean leukocyte telomere length. *PLoS One* 12:e0180687. 10.1371/journal.pone.0180687 28686726 PMC5501587

[B33] Chiu H. C. Kovacs A. Blanton R. M. Han X. Courtois M. Weinheimer C. J. (2005). Transgenic expression of fatty acid transport protein 1 in the heart causes lipotoxic cardiomyopathy. *Circ. Res.* 96 225–233. 10.1161/01.RES.0000154079.20681.B9 15618539

[B34] Chiu H. C. Kovacs A. Ford D. A. Hsu F. F. Garcia R. Herrero P. (2001). A novel mouse model of lipotoxic cardiomyopathy. *J. Clin. Invest.* 107 813–822. 10.1172/JCI10947 11285300 PMC199569

[B35] Chodari L. Mohammadi M. Ghorbanzadeh V. Dariushnejad H. Mohaddes G. (2016). Testosterone and voluntary exercise promote angiogenesis in hearts of rats with diabetes by enhancing expression of VEGF-A and SDF-1a. *Can. J. Diabetes* 40 436–441. 10.1016/j.jcjd.2016.03.004 27444229

[B36] Chong C. R. Clarke K. Levelt E. (2017). Metabolic remodeling in diabetic cardiomyopathy. *Cardiovasc. Res.* 10.1093/cvr/cvx018 [Epub ahead of print]. 28177068 PMC5412022

[B37] Clayton J. A. Collins F. S. (2014). Policy: NIH to balance sex in cell and animal studies. *Nature* 509 282–283. 10.1038/509282a 24834516 PMC5101948

[B38] Corona G. Rastrelli G. Monami M. Guay A. Buvat J. Sforza A. (2011). Hypogonadism as a risk factor for cardiovascular mortality in men: a meta-analytic study. *Eur. J. Endocrinol.* 165 687–701. 10.1530/EJE-11-0447 21852391

[B39] Crouse J. R. III (1989). Gender, lipoproteins, diet, and cardiovascular risk. Sauce for the goose may not be sauce for the gander. *Lancet* 1 318–320. 10.1016/S0140-6736(89)91320-2 2563468

[B40] Dandamudi S. Slusser J. Mahoney D. W. Redfield M. M. Rodeheffer R. J. Chen H. H. (2014). The prevalence of diabetic cardiomyopathy: a population-based study in Olmsted County, Minnesota. *J. Card. Fail.* 20 304–309. 10.1016/j.cardfail.2014.02.007 24576788 PMC4076144

[B41] Davis I. C. Ahmadizadeh I. Randell J. Younk L. Davis S. N. (2017). Understanding the impact of hypoglycemia on the cardiovascular system. *Expert Rev. Endocrinol. Metab.* 12 21–33. 10.1080/17446651.2017.1275960 29109754 PMC5669378

[B42] Dean S. A. Tan J. O’Brien E. R. Leenen F. H. (2005). 17beta-estradiol downregulates tissue angiotensin-converting enzyme and ANG II type 1 receptor in female rats. *Am. J. Physiol. Regul. Integr. Comp. Physiol.* 288 R759–R766. 10.1152/ajpregu.00595.2004 15550614

[B43] Diaz-Castro J. Pulido-Moran M. Moreno-Fernandez J. Kajarabille N. de Paco C. Garrido-Sanchez M. (2016). Gender specific differences in oxidative stress and inflammatory signaling in healthy term neonates and their mothers. *Pediatr. Res.* 80 595–601. 10.1038/pr.2016.112 27331351

[B44] Didangelos T. P. Arsos G. A. Karamitsos D. T. Athyros V. G. Karatzas N. D. (2003). Left ventricular systolic and diastolic function in normotensive type 1 diabetic patients with or without autonomic neuropathy: a radionuclide ventriculography study. *Diabetes Care* 26 1955–1960. 10.2337/diacare.26.7.1955 12832295

[B45] Emmez H. Kardes O. Dogulu F. Kurt G. Memis L. Baykaner M. K. (2008). Role of antifibrotic cytokine interferon-gamma in the prevention of postlaminectomy peridural fibrosis in rats. *Neurosurgery* 62 1351–1357; discussion 1357–1358. 10.1227/01.neu.0000333307.02802.04 18825002

[B46] Feldman H. A. Longcope C. Derby C. A. Johannes C. B. Araujo A. B. Coviello A. D. (2002). Age trends in the level of serum testosterone and other hormones in middle-aged men: longitudinal results from the Massachusetts male aging study. *J. Clin. Endocrinol. Metab.* 87 589–598. 10.1210/jcem.87.2.8201 11836290

[B47] Flanagan D. E. Holt R. I. Owens P. C. Cockington R. J. Moore V. M. Robinson J. S. (2006). Gender differences in the insulin-like growth factor axis response to a glucose load. *Acta Physiol.* 187 371–378. 10.1111/j.1748-1716.2006.01581.x 16776662

[B48] Florijn B. W. Bijkerk R. van der Veer E. van Zonneveld A. J. (2017). Gender and cardiovascular disease: are sex-biased miRNA networks a driving force behind heart failure with preserved ejection fraction in women? *Cardiovasc. Res.* 114 210–225. 10.1093/cvr/cvx223 29186452

[B49] Folsom A. R. Szklo M. Stevens J. Liao F. Smith R. Eckfeldt J. H. (1997). A prospective study of coronary heart disease in relation to fasting insulin, glucose, and diabetes. The Atherosclerosis Risk in Communities (ARIC) Study. *Diabetes Care* 20 935–942. 10.2337/diacare.20.6.935 9167103

[B50] Fontes-Carvalho R. Ladeiras-Lopes R. Bettencourt P. Leite-Moreira A. Azevedo A. (2015). Diastolic dysfunction in the diabetic continuum: association with insulin resistance, metabolic syndrome and type 2 diabetes. *Cardiovasc. Diabetol.* 14:4. 10.1186/s12933-014-0168-x 25582424 PMC4298953

[B51] Fuentes-Antras J. Picatoste B. Gomez-Hernandez A. Egido J. Tunon J. Lorenzo O. (2015). Updating experimental models of diabetic cardiomyopathy. *J. Diabetes Res.* 2015 656795. 10.1155/2015/656795 25973429 PMC4417999

[B52] Galgani J. E. Heilbronn L. K. Azuma K. Kelley D. E. Albu J. B. Pi-Sunyer X. (2008). Metabolic flexibility in response to glucose is not impaired in people with type 2 diabetes after controlling for glucose disposal rate. *Diabetes Metab. Res. Rev.* 57 841–845. 10.2337/db08-0043 18285553 PMC2756651

[B53] Gargett C. E. Bucak K. Zaitseva M. Chu S. Taylor N. Fuller P. J. (2002). Estrogen receptor-alpha and -beta expression in microvascular endothelial cells and smooth muscle cells of myometrium and leiomyoma. *Mol. Hum. Reprod.* 8 770–775. 10.1093/molehr/8.8.770 12149410

[B54] Gomez-Marcos M. A. Recio-Rodriguez J. I. Gomez-Sanchez L. Agudo-Conde C. Rodriguez-Sanchez E. Maderuelo-Fernandez J. (2015). Gender differences in the progression of target organ damage in patients with increased insulin resistance: the LOD-DIABETES study. *Cardiovasc. Diabetol.* 14:132. 10.1186/s12933-015-0293-1 26427534 PMC4591592

[B55] Gordon D. J. Probstfield J. L. Garrison R. J. Neaton J. D. Castelli W. P. Knoke J. D. (1989). High-density lipoprotein cholesterol and cardiovascular disease. Four prospective American studies. *Circulation* 79 8–15. 10.1161/01.CIR.79.1.8 2642759

[B56] Grady D. Rubin S. M. Petitti D. B. Fox C. S. Black D. Ettinger B. (1992). Hormone therapy to prevent disease and prolong life in postmenopausal women. *Ann. Intern. Med.* 117 1016–1037. 10.7326/0003-4819-117-12-1016 1443971

[B57] Gray A. Feldman H. A. McKinlay J. B. Longcope C. (1991). Age, disease, and changing sex hormone levels in middle-aged men: results of the Massachusetts Male Aging Study. *J. Clin. Endocrinol. Metab.* 73 1016–1025. 10.1210/jcem-73-5-1016 1719016

[B58] Grodstein F. Stampfer M. J. Colditz G. A. Willett W. C. Manson J. E. Joffe M. (1997). Postmenopausal hormone therapy and mortality. *N. Engl. J. Med.* 336 1769–1775. 10.1056/NEJM199706193362501 9187066

[B59] Grodstein F. Stampfer M. J. Manson J. E. Colditz G. A. Willett W. C. Rosner B. (1996). Postmenopausal estrogen and progestin use and the risk of cardiovascular disease. *N. Engl. J. Med.* 335 453–461. 10.1056/NEJM199608153350701 8672166

[B60] Grueter C. E. van Rooij E. Johnson B. A. DeLeon S. M. Sutherland L. B. Qi X. (2012). A cardiac microRNA governs systemic energy homeostasis by regulation of MED13. *Cell* 149 671–683. 10.1016/j.cell.2012.03.029 22541436 PMC3340581

[B61] Gul R. Mahmood A. Luck C. Lum-Naihe K. Alfadda A. A. Speth R. C. (2015). Regulation of cardiac miR-208a, an inducer of obesity, by rapamycin and nebivolol. *Obesity* 23 2251–2259. 10.1002/oby.21227 26381051 PMC4633375

[B62] Gusso S. Pinto T. E. Baldi J. C. Robinson E. Cutfield W. S. Hofman P. L. (2012). Diastolic function is reduced in adolescents with type 1 diabetes in response to exercise. *Diabetes Care* 35 2089–2094. 10.2337/dc11-2331 22773700 PMC3447841

[B63] Hafstad A. D. Solevag G. H. Severson D. L. Larsen T. S. Aasum E. (2006). Perfused hearts from Type 2 diabetic (db/db) mice show metabolic responsiveness to insulin. *Am. J. Physiol. Heart Circ. Physiol.* 290 H1763–H1769. 10.1152/ajpheart.01063.2005 16327015

[B64] Heyden S. Heiss G. Bartel A. G. Hames C. G. (1980). Sex differences in coronary mortality among diabetics in Evans County, Georgia. *J. Chronic Dis.* 33 265–273. 10.1016/0021-9681(80)90021-1 7372764

[B65] Hilliard L. M. Jones E. S. Steckelings U. M. Unger T. Widdop R. E. Denton K. M. (2012). Sex-specific influence of angiotensin type 2 receptor stimulation on renal function: a novel therapeutic target for hypertension. *Hypertension* 59 409–414. 10.1161/HYPERTENSIONAHA.111.184986 22158645

[B66] Holscher M. E. Bode C. Bugger H. (2016). Diabetic cardiomyopathy: does the type of diabetes matter? *Int. J. Mol. Sci.* 17:2136. 10.3390/ijms17122136 27999359 PMC5187936

[B67] Huynh K. Bernardo B. C. McMullen J. R. Ritchie R. H. (2014). Diabetic cardiomyopathy: mechanisms and new treatment strategies targeting antioxidant signaling pathways. *Pharmacol. Ther.* 142 375–415. 10.1016/j.pharmthera.2014.01.003 24462787

[B68] Huynh K. Kiriazis H. Du X. J. Love J. E. Jandeleit-Dahm K. A. Forbes J. M. (2012). Coenzyme Q10 attenuates diastolic dysfunction, cardiomyocyte hypertrophy and cardiac fibrosis in the db/db mouse model of type 2 diabetes. *Diabetologia* 55 1544–1553. 10.1007/s00125-012-2495-3 22374176

[B69] Ide T. Tsutsui H. Ohashi N. Hayashidani S. Suematsu N. Tsuchihashi M. (2002). Greater oxidative stress in healthy young men compared with premenopausal women. *Arterioscler. Thromb. Vasc. Biol.* 22 438–442. 10.1161/hq0302.104515 11884287

[B70] Iorga A. Cunningham C. M. Moazeni S. Ruffenach G. Umar S. Eghbali M. (2017). The protective role of estrogen and estrogen receptors in cardiovascular disease and the controversial use of estrogen therapy. *Biol. Sex Differ.* 8:33. 10.1186/s13293-017-0152-8 29065927 PMC5655818

[B71] Jensen M. T. Sogaard P. Andersen H. U. Bech J. Hansen T. F. Galatius S. (2014). Prevalence of systolic and diastolic dysfunction in patients with type 1 diabetes without known heart disease: the Thousand & 1 Study. *Diabetologia* 57 672–680. 10.1007/s00125-014-3164-5 24449393

[B72] Kain V. Halade G. V. (2017). Metabolic and biochemical stressors in diabetic cardiomyopathy. *Front. Cardiovasc. Med.* 4:31. 10.3389/fcvm.2017.00031 28620607 PMC5449449

[B73] Kajstura J. Gurusamy N. Ogorek B. Goichberg P. Clavo-Rondon C. Hosoda T. (2010). Myocyte turnover in the aging human heart. *Circ. Res.* 107 1374–1386. 10.1161/CIRCRESAHA.110.231498 21088285

[B74] Kannel W. B. Hjortland M. Castelli W. P. (1974). Role of diabetes in congestive heart failure: the Framingham study. *Am. J. Cardiol.* 34 29–34. 10.1016/0002-9149(74)90089-7 4835750

[B75] Kannel W. B. McGee D. L. (1979). Diabetes and cardiovascular disease. The Framingham study. *JAMA* 241 2035–2038. 10.1001/jama.1979.03290450033020 430798

[B76] Karas R. H. Patterson B. L. Mendelsohn M. E. (1994). Human vascular smooth muscle cells contain functional estrogen receptor. *Circulation* 89 1943–1950. 10.1161/01.CIR.89.5.1943 8181116

[B77] Karas R. H. Schulten H. Pare G. Aronovitz M. J. Ohlsson C. Gustafsson J. A. (2001). Effects of estrogen on the vascular injury response in estrogen receptor alpha, beta (double) knockout mice. *Circ. Res.* 89 534–539. 10.1161/hh1801.097239 11557741

[B78] Kautzky-Willer A. Harreiter J. Pacini G. (2016). Sex and gender differences in risk, pathophysiology and complications of Type 2 diabetes mellitus. *Endocr. Rev.* 37 278–316. 10.1210/er.2015-1137 27159875 PMC4890267

[B79] Kim J. K. Pedram A. Razandi M. Levin E. R. (2006). Estrogen prevents cardiomyocyte apoptosis through inhibition of reactive oxygen species and differential regulation of p38 kinase isoforms. *J. Biol. Chem.* 281 6760–6767. 10.1074/jbc.M511024200 16407188

[B80] Kloner R. A. Carson C. III Dobs A. Kopecky S. Mohler E. R. III (2016). Testosterone and cardiovascular disease. *J. Am. Coll. Cardiol.* 67 545–557. 10.1016/j.jacc.2015.12.005 26846952

[B81] Lantin-Hermoso R. L. Rosenfeld C. R. Yuhanna I. S. German Z. Chen Z. Shaul P. W. (1997). Estrogen acutely stimulates nitric oxide synthase activity in fetal pulmonary artery endothelium. *Am. J. Physiol.* 273(1 Pt 1) L119–L126. 10.1152/ajplung.1997.273.1.L119 9252548

[B82] Laverty A. A. Bottle A. Kim S. H. Visani B. Majeed A. Millett C. (2017). Gender differences in hospital admissions for major cardiovascular events and procedures in people with and without diabetes in England: a nationwide study 2004-2014. *Cardiovasc. Diabetol.* 16 1–13. 10.1186/s12933-017-0580-0 28797259 PMC5553990

[B83] Leri A. Malhotra A. Liew C. C. Kajstura J. Anversa P. (2000). Telomerase activity in rat cardiac myocytes is age and gender dependent. *J. Mol. Cell. Cardiol.* 32 385–390. 10.1006/jmcc.1999.1084 10731438

[B84] Li Z. Wang Z. Yin Z. Zhang Y. Xue X. Han J. (2017). Gender differences in fibrosis remodeling in patients with long-standing persistent atrial fibrillation. *Oncotarget* 8 53714–53729. 10.18632/oncotarget.16342 28881845 PMC5581144

[B85] Liebow I. M. Hellerstein H. K. (1949). Cardiac complications of diabetes mellitus. *Am. J. Med.* 7 660–670. 10.1016/0002-9343(49)90388-5 15396068

[B86] Logue J. Walker J. J. Colhoun H. M. Leese G. P. Lindsay R. S. McKnight J. A. (2011). Do men develop type 2 diabetes at lower body mass indices than women? *Diabetologia* 54 3003–3006. 10.1007/s00125-011-2313-3 21959958 PMC4220585

[B87] Lorenzo-Almoros A. Tunon J. Orejas M. Cortes M. Egido J. Lorenzo O. (2017). Diagnostic approaches for diabetic cardiomyopathy. *Cardiovasc. Diabetol.* 16:28. 10.1186/s12933-017-0506-x 28231848 PMC5324262

[B88] Lotufo P. A. Gaziano J. M. Chae C. U. Ajani U. A. Moreno-John G. Buring J. E. (2001). Diabetes and all-cause and coronary heart disease mortality among US male physicians. *Arch. Intern. Med.* 161 242–247. 10.1001/archinte.161.2.242 11176738

[B89] Luck C. DeMarco V. G. Mahmood A. Gavini M. P. Pulakat L. (2017). Differential regulation of cardiac function and intracardiac cytokines by rapamycin in healthy and diabetic rats. *Oxid. Med. Cell Longev.* 2017:5724046. 10.1155/2017/5724046 28408970 PMC5376943

[B90] Lum-Naihe K. T. Toedebusch R. Mahmood A. Bajwa J. Carmack T. Kumar S. (2017). Cardiovascular disease progression in female Zucker Diabetic Fatty rats occurs via unique mechanisms compared to males. *Sci. Rep.* 7:17823. 10.1038/s41598-017-18003-8 29259233 PMC5736602

[B91] Martin-Timon I. Sevillano-Collantes C. Segura-Galindo A. Del Canizo-Gomez F. J. (2014). Type 2 diabetes and cardiovascular disease: have all risk factors the same strength? *World J. Diabetes* 5 444–470. 10.4239/wjd.v5.i4.444 25126392 PMC4127581

[B92] Matarrese P. Colasanti T. Ascione B. Margutti P. Franconi F. Alessandri C. (2011). Gender disparity in susceptibility to oxidative stress and apoptosis induced by autoantibodies specific to RLIP76 in vascular cells. *Antioxid. Redox Signal.* 15 2825–2836. 10.1089/ars.2011.3942 21671802

[B93] Mazumder P. K. O’Neill B. T. Roberts M. W. Buchanan J. Yun U. J. Cooksey R. C. (2004). Impaired cardiac efficiency and increased fatty acid oxidation in insulin-resistant ob/ob mouse hearts. *Diabetes Metab. Res. Rev.* 53 2366–2374. 10.2337/diabetes.53.9.2366 15331547

[B94] Mendelsohn M. E. (2002). Protective effects of estrogen on the cardiovascular system. *Am. J. Cardiol.* 89 12E–17E; discussion 17E–18E 10.1016/S0002-9149(02)02405-0 12084397

[B95] Mendelsohn M. E. Karas R. H. (1994). Estrogen and the blood vessel wall. *Curr. Opin. Cardiol.* 9 619–626. 10.1097/00001573-199409000-00018 7987043

[B96] Mendelsohn M. E. Karas R. H. (1999). The protective effects of estrogen on the cardiovascular system. *N. Engl. J. Med.* 340 1801–1811. 10.1056/NEJM199906103402306 10362825

[B97] Miki T. Yuda S. Kouzu H. Miura T. (2013). Diabetic cardiomyopathy: pathophysiology and clinical features. *Heart Fail. Rev.* 18 149–166. 10.1007/s10741-012-9313-3 22453289 PMC3593009

[B98] Miller V. M. Best P. J. (2011). Implications for reproductive medicine: sex differences in cardiovascular disease. *Sex. Reprod. Menopause* 9 21–28. 21909244 PMC3169849

[B99] Mozaffarian D. Benjamin E. J. Go A. S. Arnett D. K. Blaha M. J. Cushman M. (2015). Heart disease and stroke statistics–2015 update: a report from the American Heart Association. *Circulation* 131 e29–322. 10.1161/CIR.0000000000000152 25520374

[B100] Mulder G. B. Luo S. Gramlich P. (2010). *The Zucker Diabetic Fatty (ZDF) Rat Diet Evaluation for the Induction of Type 2 Diabetes in Obese Female ZDF Rats.* Available at: http://www.criver.com/files/pdfs/rms/zdf/rm_rm_r_zdf_diet_eval_tech_sheet.aspx

[B101] Natarajan S. Liao Y. Cao G. Lipsitz S. R. McGee D. L. (2003). Sex differences in risk for coronary heart disease mortality associated with diabetes and established coronary heart disease. *Arch. Intern. Med.* 163 1735–1740. 10.1001/archinte.163.14.1735 12885690

[B102] Nilsson S. Gustafsson J. A. (2011). Estrogen receptors: therapies targeted to receptor subtypes. *Clin. Pharmacol. Ther.* 89 44–55. 10.1038/clpt.2010.226 21124311

[B103] Norhammar A. Schenck-Gustafsson K. (2013). Type 2 diabetes and cardiovascular disease in women. *Diabetologia* 56 1–9. 10.1007/s00125-012-2694-y 22945305

[B104] Okumura M. Iwai M. Ide A. Mogi M. Ito M. Horiuchi M. (2005). Sex difference in vascular injury and the vasoprotective effect of valsartan are related to differential AT2 receptor expression. *Hypertension* 46 577–583. 10.1161/01.HYP.0000178564.14464.80 16103268

[B105] Oldroyd S. D. Thomas G. L. Gabbiani G. El Nahas A. M. (1999). Interferon-gamma inhibits experimental renal fibrosis. *Kidney Int.* 56 2116–2127. 10.1046/j.1523-1755.1999.00775.x 10594787

[B106] Olivetti G. Giordano G. Corradi D. Melissari M. Lagrasta C. Gambert S. R. (1995). Gender differences and aging: effects on the human heart. *J. Am. Coll. Cardiol.* 26 1068–1079. 10.1016/0735-1097(95)00282-8 7560601

[B107] Patten R. D. Pourati I. Aronovitz M. J. Baur J. Celestin F. Chen X. (2004). 17beta-estradiol reduces cardiomyocyte apoptosis in vivo and in vitro via activation of phospho-inositide-3 kinase/Akt signaling. *Circ. Res.* 95 692–699. 10.1161/01.RES.0000144126.57786.89 15345655

[B108] Perseghin G. Ntali G. De Cobelli F. Lattuada G. Esposito A. Belloni E. (2007). Abnormal left ventricular energy metabolism in obese men with preserved systolic and diastolic functions is associated with insulin resistance. *Diabetes Care* 30 1520–1526. 10.2337/dc06-2429 17384336

[B109] Peters S. A. Huxley R. R. Woodward M. (2014). Diabetes as risk factor for incident coronary heart disease in women compared with men: a systematic review and meta-analysis of 64 cohorts including 858,507 individuals and 28,203 coronary events. *Diabetologia* 57 1542–1551. 10.1007/s00125-014-3260-6 24859435

[B110] Peterson L. R. Herrero P. Schechtman K. B. Racette S. B. Waggoner A. D. Kisrieva-Ware Z. (2004). Effect of obesity and insulin resistance on myocardial substrate metabolism and efficiency in young women. *Circulation* 109 2191–2196. 10.1161/01.CIR.0000127959.28627.F8 15123530

[B111] Pulakat L. DeMarco V. G. Ardhanari S. Chockalingam A. Gul R. Whaley-Connell A. (2011). Adaptive mechanisms to compensate for overnutrition-induced cardiovascular abnormalities. *Am. J. Physiol. Regul. Integr. Comp. Physiol.* 301 R885–R895. 10.1152/ajpregu.00316.2011 21813874 PMC3289980

[B112] Qi Y. Li H. Shenoy V. Li Q. Wong F. Zhang L. (2012). Moderate cardiac-selective overexpression of angiotensin II type 2 receptor protects cardiac functions from ischaemic injury. *Exp. Physiol.* 97 89–101. 10.1113/expphysiol.2011.060673 21967903 PMC3619662

[B113] Romanens M. Fankhauser S. Saner B. Michaud L. Saner H. (1999). No evidence for systolic or diastolic left ventricular dysfunction at rest in selected patients with long-term type I diabetes mellitus. *Eur. J. Heart Fail.* 1 169–175. 10.1016/S1388-9842(99)00012-4 10937927

[B114] Rosano G. M. Leonardo F. Pagnotta P. Pelliccia F. Panina G. Cerquetani E. (1999). Acute anti-ischemic effect of testosterone in men with coronary artery disease. *Circulation* 99 1666–1670. 10.1161/01.CIR.99.13.1666 10190874

[B115] Rubler S. Dlugash J. Yuceoglu Y. Z. Kumral T. Branwood A. W. Grishman A. (1972). New type of cardiomyopathy associated with diabetic glomerulosclerosis. *Am. J. Cardiol.* 30 595–602. 10.1016/0002-9149(72)90595-4 4263660

[B116] Ruige J. B. Mahmoud A. M. De Bacquer D. Kaufman J. M. (2011). Endogenous testosterone and cardiovascular disease in healthy men: a meta-analysis. *Heart* 97 870–875. 10.1136/hrt.2010.210757 21177660

[B117] Salinero A. E. Anderson B. M. Zuloaga K. L. (2018). Sex differences in the metabolic effects of diet-induced obesity vary by age of onset. *Int. J. Obes.* 10.1038/s41366-018-0023-3 [Epub ahead of print]. 29463918

[B118] Sampson A. K. Moritz K. M. Jones E. S. Flower R. L. Widdop R. E. Denton K. M. (2008). Enhanced angiotensin II type 2 receptor mechanisms mediate decreases in arterial pressure attributable to chronic low-dose angiotensin II in female rats. *Hypertension* 52 666–671. 10.1161/HYPERTENSIONAHA.108.114058 18711010

[B119] Sandoo A. van Zanten J. J. Metsios G. S. Carroll D. Kitas G. D. (2010). The endothelium and its role in regulating vascular tone. *Open Cardiovasc. Med. J.* 4 302–312. 10.2174/1874192401004010302 21339899 PMC3040999

[B120] Sawhney V. Brouilette S. Campbell N. Coppen S. Baker V. Hunter R. (2018). Association of genetic variation in telomere-related SNP and telomerase with ventricular arrhythmias in ischemic cardiomyopathy. *Pacing Clin. Electrophysiol.* 10.1111/pace.13284 [Epub ahead of print]. 29344960

[B121] Schilling J. D. Mann L. D. (2014). Diabetic cardiomyopathy: distinct and preventable entity or inevitable consequence? *Curr. Cariovasc. Risk Rep.* 8:417 10.1007/s12170-014-0417-2

[B122] Semeniuk L. M. Kryski A. J. Severson D. L. (2002). Echocardiographic assessment of cardiac function in diabetic db/db and transgenic db/db-hGLUT4 mice. *Am. J. Physiol. Heart Circ. Physiol.* 283 H976–H982. 10.1152/ajpheart.00088.2002 12181126

[B123] Severson D. L. (2004). Diabetic cardiomyopathy: recent evidence from mouse models of type 1 and type 2 diabetes. *Can. J. Physiol. Pharmacol.* 82 813–823. 10.1139/y04-065 15573141

[B124] Sharma S. Adrogue J. V. Golfman L. Uray I. Lemm J. Youker K. (2004). Intramyocardial lipid accumulation in the failing human heart resembles the lipotoxic rat heart. *FASEB J.* 18 1692–1700. 10.1096/fj.04-2263com 15522914

[B125] Shi J. Li J. Guan H. Cai W. Bai X. Fang X. (2014). Anti-fibrotic actions of interleukin-10 against hypertrophic scarring by activation of PI3K/AKT and STAT3 signaling pathways in scar-forming fibroblasts. *PLoS One* 9:e98228. 10.1371/journal.pone.0098228 24878845 PMC4039501

[B126] Simons L. A. McCallum J. Friedlander Y. Simons J. (1996). Diabetes, mortality and coronary heart disease in the prospective Dubbo study of Australian elderly. *Aust. N. Z. J. Med.* 26 66–74. 10.1111/j.1445-5994.1996.tb02909.x 8775531

[B127] Skorska A. von Haehling S. Ludwig M. Lux C. A. Gaebel R. Kleiner G. (2015). The CD4(+) AT2R(+) T cell subpopulation improves post-infarction remodelling and restores cardiac function. *J. Cell. Mol. Med.* 19 1975–1985. 10.1111/jcmm.12574 25991381 PMC4549048

[B128] Spark R. F. (2007). Testosterone, diabetes mellitus, and the metabolic syndrome. *Curr. Urol. Rep.* 8 467–471. 10.1007/s11934-007-0050-4 18042326

[B129] Stahrenberg R. Edelmann F. Mende M. Kockskamper A. Dungen H. D. Scherer M. (2010). Association of glucose metabolism with diastolic function along the diabetic continuum. *Diabetologia* 53 1331–1340. 10.1007/s00125-010-1718-8 20386878 PMC2877336

[B130] Stanley W. C. Lopaschuk G. D. McCormack J. G. (1997). Regulation of energy substrate metabolism in the diabetic heart. *Cardiovasc. Res.* 34 25–33. 10.1016/S0008-6363(97)00047-3 9217869

[B131] Suran D. Sinkovic A. Naji F. (2016). Tissue Doppler imaging is a sensitive echocardiographic technique to detect subclinical systolic and diastolic dysfunction of both ventricles in type 1 diabetes mellitus. *BMC Cardiovasc. Disord.* 16:72. 10.1186/s12872-016-0242-2 27102111 PMC4840968

[B132] Varma U. Koutsifeli P. Benson V. L. Mellor K. M. Delbridge L. M. D. (2017). Molecular mechanisms of cardiac pathology in diabetes - Experimental insights. *Biochim. Biophys. Acta* 1864 1949–1959. 10.1016/j.bbadis.2017.10.035 29109032

[B133] Venkov C. D. Rankin A. B. Vaughan D. E. (1996). Identification of authentic estrogen receptor in cultured endothelial cells. A potential mechanism for steroid hormone regulation of endothelial function. *Circulation* 94 727–733. 10.1161/01.CIR.94.4.727 8772695

[B134] Vrtacnik P. Ostanek B. Mencej-Bedrac S. Marc J. (2014). The many faces of estrogen signaling. *Biochem. Med.* 24 329–342. 10.11613/BM.2014.035 25351351 PMC4210253

[B135] Wachter R. Luers C. Kleta S. Griebel K. Herrmann-Lingen C. Binder L. (2007). Impact of diabetes on left ventricular diastolic function in patients with arterial hypertension. *Eur. J. Heart Fail.* 9 469–476. 10.1016/j.ejheart.2007.01.001 17303471

[B136] Wang Y. Cao Y. Yamada S. Thirunavukkarasu M. Nin V. Joshi M. (2015). Cardiomyopathy and worsened ischemic heart failure in SM22-alpha Cre-mediated neuropilin-1 null mice: dysregulation of PGC1alpha and mitochondrial homeostasis. *Arterioscler. Thromb. Vasc. Biol.* 35 1401–1412. 10.1161/ATVBAHA.115.305566 25882068 PMC4441604

[B137] Wannamethee S. G. Papacosta O. Lawlor D. A. Whincup P. H. Lowe G. D. Ebrahim S. (2012). Do women exhibit greater differences in established and novel risk factors between diabetes and non-diabetes than men? The British Regional Heart Study and British Women’s Heart Health Study. *Diabetologia* 55 80–87. 10.1007/s00125-011-2284-4 21861177

[B138] Weiner C. P. Lizasoain I. Baylis S. A. Knowles R. G. Charles I. G. Moncada S. (1994). Induction of calcium-dependent nitric oxide synthases by sex hormones. *Proc. Natl. Acad. Sci. U.S.A.* 91 5212–5216. 10.1073/pnas.91.11.5212 7515189 PMC43962

[B139] Widdop R. E. Jones E. S. Hannan R. E. Gaspari T. A. (2003). Angiotensin AT2 receptors: cardiovascular hope or hype? *Br. J. Pharmacol.* 140 809–824. 10.1038/sj.bjp.0705448 14530223 PMC1574085

[B140] Wilson M. S. Elnekave E. Mentink-Kane M. M. Hodges M. G. Pesce J. T. Ramalingam T. R. (2007). IL-13Ralpha2 and IL-10 coordinately suppress airway inflammation, airway-hyperreactivity, and fibrosis in mice. *J. Clin. Invest.* 117 2941–2951. 10.1172/JCI31546 17885690 PMC1978425

[B141] World Health Organization [WHO] (2017). *Cardiovascular Diseases (CVD) [Online].* Geneva: World Health Organization.

[B142] Wu Y. Cazorla O. Labeit D. Labeit S. Granzier H. (2000). Changes in titin and collagen underlie diastolic stiffness diversity of cardiac muscle. *J. Mol. Cell Cardiol.* 32 2151–2162. 10.1006/jmcc.2000.1281 11112991

[B143] Wu Y. Shen Y. Kang K. Zhang Y. Ao F. Wan Y. (2015). Effects of estrogen on growth and smooth muscle differentiation of vascular wall-resident CD34(+) stem/progenitor cells. *Atherosclerosis* 240 453–461. 10.1016/j.atherosclerosis.2015.04.008 25898000

[B144] Xu Y. Arenas I. A. Armstrong S. J. Davidge S. T. (2003). Estrogen modulation of left ventricular remodeling in the aged heart. *Cardiovasc. Res.* 57 388–394. 10.1016/S0008-6363(02)00705-8 12566111

[B145] Yang X. P. Reckelhoff J. F. (2011). Estrogen, hormonal replacement therapy and cardiovascular disease. *Curr. Opin. Nephrol. Hypertens.* 20 133–138. 10.1097/MNH.0b013e3283431921 21178615 PMC3123884

[B146] Yeh J. K. Wang C. Y. (2016). Telomeres and telomerase in cardiovascular diseases. *Genes* 7:E58. 10.3390/genes7090058 27598203 PMC5042389

[B147] Yen S. S. C. Jade R. B. (eds) (1991). *Reproductive Endocrinology: Physiology, Pathophysiology and Clinical Management.* Philadelphia, PA: W.B. Saunders.

[B148] Zarich S. W. Arbuckle B. E. Cohen L. R. Roberts M. Nesto R. W. (1988). Diastolic abnormalities in young asymptomatic diabetic patients assessed by pulsed Doppler echocardiography. *J. Am. Coll. Cardiol.* 12 114–120. 10.1016/0735-1097(88)90364-6 3379197

[B149] Zhang B. Shen Q. Chen Y. Pan R. Kuang S. Liu G. (2017). Myricitrin alleviates oxidative stress-induced inflammation and apoptosis and protects mice against diabetic cardiomyopathy. *Sci. Rep.* 7:44239. 10.1038/srep44239 28287141 PMC5347164

[B150] Zhang H. Morgan B. Potter B. J. Ma L. Dellsperger K. C. Ungvari Z. (2010). Resveratrol improves left ventricular diastolic relaxation in type 2 diabetes by inhibiting oxidative/nitrative stress: in vivo demonstration with magnetic resonance imaging. *Am. J. Physiol. Heart Circ. Physiol.* 299 H985–H994. 10.1152/ajpheart.00489.2010 20675566 PMC2957362

[B151] Zhao J. Imbrie G. A. Baur W. E. Iyer L. K. Aronovitz M. J. Kershaw T. B. (2013). Estrogen receptor-mediated regulation of microRNA inhibits proliferation of vascular smooth muscle cells. *Arterioscler. Thromb. Vasc. Biol.* 33 257–265. 10.1161/ATVBAHA.112.300200 23175673 PMC3780598

[B152] Zhao R. Le K. Moghadasian M. H. Shen G. X. (2013). Regulatory role of NADPH oxidase in glycated LDL-induced upregulation of plasminogen activator inhibitor-1 and heat shock factor-1 in mouse embryo fibroblasts and diabetic mice. *Free Radic. Biol. Med.* 61 18–25. 10.1016/j.freeradbiomed.2013.03.009 23511120

[B153] Zucker I. Beery A. K. (2010). Males still dominate animal studies. *Nature* 465:690. 10.1038/465690a 20535186

